# Angiogenesis as a Survival Mechanism in Heartworm Disease: The Role of Fructose-Bisphosphate Aldolase and Actin from *Dirofilaria immitis* in an In Vitro Endothelial Model

**DOI:** 10.3390/ani14233371

**Published:** 2024-11-22

**Authors:** Manuel Collado-Cuadrado, Claudia Alarcón-Torrecillas, Alfonso Balmori-de la Puente, Iván Rodríguez-Escolar, Elena Infante González-Mohino, Miguel Pericacho, Rodrigo Morchón

**Affiliations:** 1 Zoonotic Diseases and One Health Group, Faculty of Pharmacy, Biomedical Research Institute of Salamanca (IBSAL), Centre for Environmental Studies and Rural Dynamization (CEADIR), University of Salamanca, 37007 Salamanca, Spain; manuelcollado@usal.es (M.C.-C.); a.balmori@usal.es (A.B.-d.l.P.); ivanrodriguez@usal.es (I.R.-E.); elena.igm4@usal.es (E.I.G.-M.); 2 Department of Physiology and Pharmacology, Biomedical Research Institute of Salamanca (IBSAL), University of Salamanca, 37007 Salamanca, Spain; claudia3alarcon@usal.es (C.A.-T.); pericacho@usal.es (M.P.)

**Keywords:** angiogenesis, *Dirofilaria immitis* excretory/secretory antigen, actin, fructose-bisphosphate aldolase, VEGF-A, sVEGFR-2, mEndoglin, cellular proliferation, cellular migration, pseudocapillary formation

## Abstract

Heartworm disease, caused by *Dirofilaria immitis,* is a vector-borne zoonotic disease, (mainly affecting canids and felids) causing chronic vascular and pulmonary pathology in its early stages, which worsens with parasite load and/or death of adult worms in the pulmonary artery or right heart cavity, and can be fatal to the host. Angiogenesis is a mechanism by which new blood vessels are formed from existing ones. The aim of this work was to study the effect of two molecules of the *D. immitis* excretory/secretory antigen (DiES) on the angiogenic process, taking into account that this antigen is able to interact with this process and use it as a survival mechanism. For this purpose, an in vitro model of endothelial cells was used and treated with two recombinant proteins, i.e., actin (Act) and fructose-bisphosphate aldolase (FBAL) proteins belonging to DiES, and both pro- and antiangiogenic molecules were analyzed, as well as the cellular processes of cell proliferation, migration, and pseudocapillary formation. Act and FBAL proteins, together with vascular endothelial growth factor (VEGF-A), as an angiogenic precursor, are able to stimulate the production of proangiogenic factors as well as cellular processes of proliferation, migration, and pseudocapillary formation. This implies that these molecules could be produced by *D. immitis* to facilitate its survival, and the relationship between parasite and canine host would be further elaborated.

## Simple Summary

Cardiopulmonary dirofilariosis is a chronic and potentially fatal vascular and pulmonary disease caused by *Dirofilaria immitis*, mainly affecting canids and felids. The disease is exacerbated by the production of thrombi following the death of the adult worms naturally or by adulticidal treatment. However, *D. immitis* is able to live for years in the host. Its excretory/secretory antigen (DiES) has been shown to interact with angiogenesis, a mechanism by which the host is able to form new blood vessels from existing ones. Our aim was to analyze the effect of the recombinant proteins actin (rDiAct) and fructose-bisphosphate aldolase (rDiFBAL), belonging to DiES, together with VEGF-A (angiogenic precursor) on the angiogenic process in an in vitro model of endothelial cells. The production of both pro- and antiangiogenic molecules was analyzed, as well as the cellular processes of cell proliferation and migration, and the formation of pseudocapillaries. Both rDiAct + VEGF-A and rDiFBAL + VEGF-A stimulate proangiogenic and cellular processes associated with the proangiogenic process. These results seem to indicate that *D. immitis* is able to stimulate specific molecules within its excretory/secretory antigen that aids its survival in the host vascular system.

## 1. Introduction

Cardiopulmonary dirofilariosis is a zoonotic disease for which the causative agent is *Dirofilaria immitis*, and which is transmitted by culicid vectors of the genera *Culex* spp., *Aedes* spp., and *Anoplehes* spp., among others [1]. It is a vascular and pulmonary disease that mainly affects canids and felids. The latter are hosts that are not as adapted to the disease as are dogs since, when infected, they often develop HARD syndrome in the first months, which differs from the classic form of heartworm disease. However, in dogs, *D. immitis* causes a severe and potentially fatal pathology, with a chronic and progressive course, whose most important damage is caused by the presence of *D. immitis* adult worms in the pulmonary artery and in the right ventricle of the heart of the infected animal. The most important damages include proliferative endoarteritis, pulmonary edema and hypertension, and potential congestive heart failure [2,3,4]. In addition, acute processes may appear due to the death of *D. immitis* adult worms, which increase the generation of thromboembolisms of severe character, which aggravate vascular and pulmonary damage, and increase the probability of congestive heart failure, being fatal for the infected animal [5]. Not only are the vascular and pulmonary systems affected, but also we can find cases with membranous glomerulonephritis, presence of microfilariae in the nephritic glomeruli, proteinuria in urine in infected dogs, and even ectopic locations such as in the ocular area, subcutaneous area, testicles, abdomen, etc. [6,7].

The mechanical friction of *D. immitis* on the vascular endothelium and the presence of *Wolbachia* sp. (endosymbiont bacterium of *D. immitis*) produce loss of elasticity and increased permeability in the vessel wall, increased intercellular spaces, degradation of the elastic layer, and stimulation of smooth muscle cells, favoring the formation of vascular villi generating proliferative endoarteritis and outflow of fluid to the pulmonary parenchyma, which is the origin of pulmonary edema [8]. In addition, the accumulation of adult *D. immitis* worms in the pulmonary artery generates luminal obstruction, arterial hypertension with reduced blood flow, and a hypoxic situation (lack of oxygen), which can be prolonged in time, since the adult worms can live for years in the infected animal [8,9,10,11].

One process that is related to this condition is angiogenesis [12], a process by which new blood vessels are formed from existing ones. This mechanism is essential in several physiological processes such as wound healing, embryonic development, and tissue regeneration, but also plays a key role in several diseases that induce the formation of new blood vessels to supply nutrients and oxygen [13]. In response to stimuli such as hypoxia, cells produce proangiogenic growth factors, with the vascular endothelial growth factor (VEGF) being the most important [14]. Growth factors such as VEGF bind to their specific receptors on the surface of endothelial cells, which are the cells that form the inner walls of blood vessels (VEGFR). Endothelial cells activated by binding to VEFGR-2 produce soluble VEGFR-2 (sVEGFR-2) and enzymes, such as matrix metalloproteinases, that degrade the surrounding extracellular matrix, allowing the endothelial cells to migrate and proliferate. Endothelial cells migrate to the area where new vessel formation is required [15]. They also multiply to extend the vascular network. Endothelial cells organize their structure forming tubules that eventually transform into functional vessels [16,17]. This process can be counteracted by binding to VEGFR-1 receptors and its soluble form sFlt1, which exerts an angiogenic negative regulatory effect [18,19]. Other key molecules in the angiogenic pathway are membrane endoglin (mEndoglin) that activates cell proliferation, while soluble endoglin (sEndoglin) is activated by metalloproteinases, which promote nonangiogenic process and/or endothelial vasoconstriction [20,21].

The excretory/secretory antigen of *D. immitis* and its surface antigen are capable of regulating the angiogenic process. On the one hand, they are capable of promoting the proangiogenic pathway, prior to the production of VEGF-A, and, on the other hand, stimulating cell proliferation and migration and the formation of pseudocapillaries [22]. Similarly, other parasites, such as *D. repens*, are capable of activating the proangiogenic pathway as a survival mechanism [23], while Wolbachia sp. regulates the antiangiogenic action, while promoting the proinflammatory response, and generating damage in the definitive host [24,25,26].

The aim of this study was to individualize the role of actin (rDiACT) and fructose-bisphosphate aldolase (rDiFBAL) recombinant proteins, located in the excretory/secretory and surface antigen of *D. immitis* adult worms, respectively [27,28], in the angiogenic process and cellular processes related to the proangiogenic pathway.

## 2. Materials and Methods

### 2.1. Antigens and Molecules Used

*Dirofilaria immitis* excretory/secretory antigen (DiES) was prepared according to the methodology previously described by González-Miguel et al. [27] with some modifications. Twenty-seven live *D. immitis* adult worms from the same dog (6 males and 21 females) were washed in sterile PBS pH 7.2 and incubated for 24 h in 50 mL of Eagle’s Minimum Essential Medium (Sigma Chemical Company, St. Louis, MO, USA) supplemented with 0.04% gentamicin and 0.01% nystatin at 37 °C. The resulting medium was then incubated for 24 h in 50 mL of Eagle’s Minimum Essential Medium (Sigma Chemical Company, St. Louis, MO, USA) supplemented with 0.04% gentamicin and 0.01% nystatin at 37 °C. The resulting medium was then dialyzed in 0.01% PBS pH 7.2 and filtered through an Amicon YC05 (Amicon Corporation Scientific System Division, Danvers, MA, USA).

Actin (ACT) and fructose-bisphosphate aldolase (FBAL) proteins belonging to DiES and surface antigen of *D. immitis*, respectively were recombinantly produced (rDiACT and rDiFBAL) following the methodology described by González-Miguel et al. [29] with some modifications. In brief, RNA was extracted from adult *D. immitis* worms using the NucleoSpin RNA II kit (Macherey-Nagel), first-strand cDNA was synthesized using the first-strand cDNA kit (Roche), and the cDNA sequences of FBAL and Act from *D. immitis* were amplified using the following primers: ACTFwd (5′-ATGTGTGACGACGACGTTGCGG), ACTRev (5′-CTAGAAACATTTGCGATGAACAATTG), FBALFwd (5′-ATGACCTCTTACTCACAGTTTCTG), and FBALRev (5′-TTAGTATGCATGATTAGCAATGTAG). PCR amplifications were performed in 1 cycle at 94 °C for 5 m, 35 cycles at 94 °C for 1 m, 46 °C for 46 s, and 72 °C for 1 min 30 s and 1 cycle at 72 °C for 5 m. PCR products were loaded on agarose gel, and electrophoresis was performed, and the bands were purified using the StrataPrep DNA Gel Extraction Kit (Stratagene). DiFBAL and DiAct products were then cloned into the pSC-A vector using the StrataClone PCR Cloning kit (Stratagene) and purified using the Machery-Nagel NuceoSplin Plasmid kit. For expression and purification of DiAct and DiFBAL, PCR products containing the coding sequence of the proteins were cloned into the TOPO vector (Invitrogen) following the manufacturer’s instructions. Recombinant plasmids were transformed into *Escherichia coli* XL1B strains grown on LB agar plates with amplicin (100 μg/mL). Vectors containing the sequence of interest in the correct reading frame were transformed into BL-21 cells in liquid LB plus amplicin (100 μg/mL), and subsequently, protein expression was induced by 0.2% L-arabinose. Transfected cells were sonicated in a buffer containing 8 M urea, 100 mM NaH_2_PO_4_, and 10 mM Tris-Cl, pH 7.9. After a 20 min centrifugation step at 10,000× *g*, the supernatant was applied to the HIS-Select Nickel affinity gel (Sigma) for protein purification, following the manufacturer’s instructions. Subsequently, the recombinant forms of DiAct and DiFBAL were eluted in elution buffer (50 mM NaH_2_PO_4_, 300 mM NaCl, and 250 mM imidazole, pH 7.9) and dialyzed in PBS and then stored until use at −80 °C.

### 2.2. Cell Culture and Endothelial Cell Stimulation

A primary culture of human umbilical vein endothelial cells (HUVECs) was used in Endothelial Basal Medium 2 (Lonza, Walkersville, MD, USA) supplemented with SingleQuots^®^ (Lonza, Walkersville, MD, USA): 20% fetal bovine serum (22.5 µg/mL), heparin (22.5 µg/mL), VEGF (0.5 ng/mL), ascorbic acid (1 µg/mL), hFGF-B (10 ng/mL), hydrocortisone (0.2 µg/mL), hEGF (5 ng/mL), gentamicin (30 mg/mL), amphotericin B (15 µg/mL), and R^3^-IGF-3 (20 ng/mL). Plates were pre-coated with 0.1% pig gelatine (Sigma Chemical Co., St. Louis, MO, USA), 0.01% fibronectin (Sigma-Aldrich, St. Louis, MO, USA), and 0.01% collagen (Corning). Cells were cultured at 37 °C in a humidified atmosphere in the presence of 5% CO_2_/95% air. The medium was changed every 3 days. Expansion was carried out by trypsinizing the cells (Trypsin/EDTA, Lonza, USA) and placing them on the new plates when the proliferating cells had reached a sufficient density. Passaging was performed under the ratio of 1:3. Cell counts were performed using a Countess^®^ Automated Cell Counter (Invitrogen, Carlsbad, CA, USA) following the manufacturer’s instructions.

HUVECs were treated as previously described by Machado et al. [22]. In brief, endothelial cells were cultured in 60 mm dishes and allowed to grow to confluent cultures and then treated for 24 h with VEGF-A (R&D SYSTEMS), rDiACT, and rDiFBAL at 1 μg/mL and rDiACT + VEGF-A and rDiFBAL + VEGF-A at 1 μg/mL for each of the molecules used. Unstimulated cell cultures were used as negative control (Control) and cell cultures stimulated with DiES + VEGF-A (1 μg/mL each) were used as positive control (Control +) [22] Finally, the supernatant was collected, and cells were lysed using lysis buffer (20 mM Tris-HCl pH 7.5, 140 mM NaCl, 10 mM ethylenediaminetetraacetic acid, 10% glycerol, 1% Igepal CA-630, aprotinin, pepstatin, and leupeptin at 1 μg/mL each, 1 mM phenylmethlsulfonyl fluoride, and 1 mM sodium orthovanadate).

### 2.3. Cell Viability and Cytotoxicity Test

Cell viability was measured by counting cells using the Countess Automated Cell Counter (Invitrogen) according to the manufacturer’s instructions. Cytotoxicity was analyzed from the supernatant of stimulated and control cultures using the Toxilight BioAssay Kit (Cambrex, Verviers, Belgium) according to the manufacturer’s instructions. This commercial kit quantitatively measures adenylate cyclase release from damaged cells. Results are presented as the mean ± standard deviation (SD) of three experiments performed in duplicate.

### 2.4. Pro- and Antiangiogenic Factors’ Assays

VEGF-A, VEGFR-1/sFlt1, sVEGFR-2, and sEndoglin concentration in the endothelial cells culture medium and mEndoglin concentration in the endothelial cells lysed were measured by Human Quantikine ELISA kit, respectively (R&D Systems, Minneapolis, MN, USA), following the manufacturers’ instructions. The results are presented as the mean ± SD of three experiments performed in duplicates.

### 2.5. Cellular Proliferation Assay

In relation to cell proliferation, 800 cells were seeded on a 96-well plate and stimulated with the different treatments mentioned above. Unstimulated cell cultures were used as negative control, and cell cultures stimulated with DiES + VEGF-A were used as positive control in the same conditions. Proliferation at 10 days of culture was determined every 2 days by incubating cell cultures with 0.5 mg/mL 3-[4.5-dimethylthiazol-2-yl]-2.5-diphenyl tetrazolium bromide (MTT) (Sigma-Aldrich, St. Louis, MO, USA) for 4 h. Then, 10% SDS in 0.01 M HCl was added at a 1:1 (*v*/*v*) ratio and left overnight at 37 °C. Finally, absorbance was measured at 595 nm. The results are presented as the mean ± SD of three experiments.

### 2.6. Cellular Migration Assays

In vitro scratch wounds (wound healing) were created by scraping confluent cell monolayers in 35 mm sterile plates with Mitomycin (M5655, Sigma) to inhibit cell division and favor migration, and vertical wounds were made with a sterile pipette tip. The plates were then washed with sterile PBS to remove lifted or dead cells and were treated with the different stimuli for 6 h. Unstimulated cells were used as negative control, and DiES + VEGF-A-treated cells were used as positive control under the same conditions. Endothelial cell migration was monitored every 30 min with a digital camera coupled to an inverted phase contrast microscope. Results are presented as the mean ± SD of at least 3 times per experiment.

### 2.7. Pseudocapillary Formation Assays

Fifteen-well (0.23 cm^2^) Ibidi Angiogenesis Slide plates (Ibidi, Gräfelfing, Germany) previously incubated with Matrigel^®^ (356237, Corning, Glendale, AZ, USA) were used, and 7.5–9 × 10^3^ cells/well were seeded with the different treatments. Connections and cell bodies were quantified by counting their number 90 min after treatment by taking pictures with an inverted microscope. Then, intercellular connections were divided between cell bodies to calculate the ratio between them (pseudocapillary formation = cell connections/cell bodies). Unstimulated cells were used as negative control and cells stimulated with DiES + VEGF-A were used as positive control under the same conditions. Results are presented as the mean ± SD of at least 3 times per experiment.

### 2.8. Statistical Analysis

R software version 4.4 was used for all data analyses. Analyses were performed by ANOVA and corrected for repeated measurements when appropriate. If ANOVA revealed overall significant differences, individual means were evaluated post hoc using Tukey’s test. All results were expressed as the mean ± SD. In all experiments, a significant difference was defined as a *p* value (*p*) < 0.05 and <0.01.

## 3. Results

### 3.1. Effect of rDiACT and rDiFBAL on Cell Viability, Cytotoxicity, Angiogenic Factors, and Endoglin Production

No significant differences in cell viability and cytotoxicity were found between unstimulated cultures (Control) and stimulated cell cultures with DiES + VEGF-A (Control +) VEGF-A, rDiACT, rDiFBAL, rDiACT + VEGF-A, and rDiFBAL + VEGF-A.

**Figure 1:**
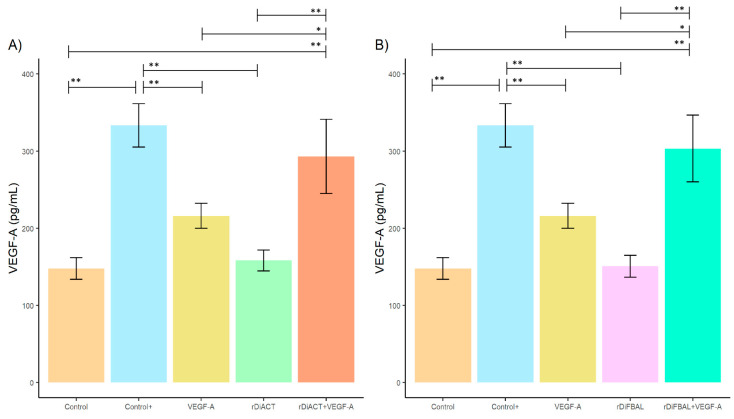
Effects of rDiACT (**A**) and rDiFBAL (**B**) on VEGF-A production in unstimulated cultures (Control) (■) and cultures stimulated with DiES + VEGF-A (Control +) (■), VEGF-A (■), rDiACT (■), rDiACT +VEGF-A (■), rDiFBAL (■), and rDiFBAL + VEGF-A (■). Results are expressed as the mean ± SD of 3 independent experiments. The asterisk (*) and double asterisk (**) indicate significant differences with *p* < 0.05 and *p* < 0.01, respectively.

In relation to the angiogenic pathway stimulation study (Figure 1), stimulation of cell cultures with rDiACT + VEGF-A resulted in a significant increase in VEGF-A production compared to cultures stimulated with rDiACT (*F* = 26.87; *df* = 4; *p* = 0.0009), VEGF-A (*F* = 26.87; *df* = 4; *p* = 0.0389), and unstimulated cultures (Control) (*F* = 26.87; *df* = 4; *p* < 0.0001) during the first 24 h. Stimulation of cell cultures with rDiFBAL + VEGF-A also resulted in a significant increase in VEGF-A production compared to cultures stimulated with rDiFBAL (*F* = 32.94; *df* = 4; *p* = 0.0021), VEGF-A (*F* = 32.94; *df* = 4; *p* = 0.0134), and unstimulated cultures (Control) (*F* = 32.94; *df* = 4; *p* = 0.0017), during the first 24 h. There were no significant differences between Control + and cultures stimulated with rDiACT + VEGF-A and rDiFBAL + VEGF-A, respectively, but there were significant differences with cultures stimulated with VEGF-A (*F* = 32.94; *df* = 4; *p* = 0.0011), rDiACT (*F* = 26.87; *df* = 4; *p* = 0.0001), and rDiFBAL (*F* = 32.94; *df* = 4; *p* < 0.0001).

**Figure 2:**
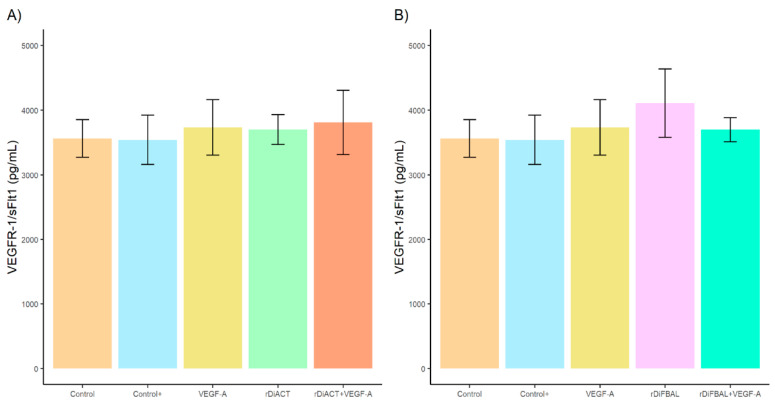
Effects of rDiACT (**A**) and rDiFBAL (**B**) on VEGFR-1/sFlt1 production in unstimulated cultures (Control) (■) and cultures stimulated with DiES + VEGF-A (Control +) (■), VEGF-A (■), rDiACT (■), rDiACT +VEGF-A (■), rDiFBAL (■), and rDiFBAL + VEGF-A (■). Results are expressed as the mean ± SD of 3 independent experiments.

**Figure 3:**
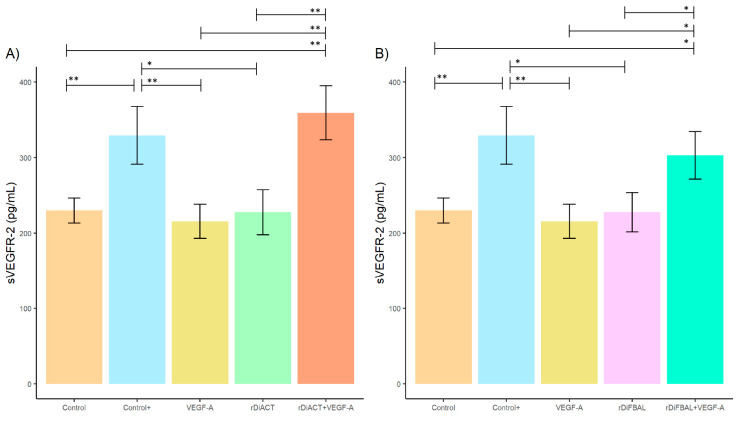
Effects of rDiACT (**A**) and rDiFBAL (**B**) on sVEGFR-2 production in unstimulated cultures (Control) (■) and cultures stimulated with DiES + VEGF-A (Control +) (■), VEGF-A (■), rDiACT (■), rDiACT +VEGF-A (■), rDiFBAL (■), and rDiFBAL + VEGF-A (■). Results are expressed as the mean ± SD of 3 independent experiments. The asterisk (*) and double asterisk (**) indicate significant differences with *p* < 0.05 and *p* < 0.01, respectively.

Regarding VEGFR-1/sFlt1 concentration, no significant differences were found between stimulated and unstimulated cultures (Figure 2). However, there was a significant increase in sVEGFR-2 concentration (Figure 3) in cultures stimulated with rDiACT + VEGF-A and rDiFBAL + VEGF-A compared to cultures stimulated with rDiACT (*F* = 15.15; *df* = 4; *p* = 0.002) and rDiFBAL (*F* = 10.14; *df* = 4; *p* = 0.0494), respectively, to cell cultures stimulated with VEGF-A (*F* = 15.15; *df* = 4; *p* = 0.001; and *F* = 10.14; *df* = 4; *p* = 0.022, respectively) and to untreated cultures (Control) (*F* = 15.15; *df* = 4; *p* = 0.0023; and *F* = 10.14; *df* = 4; *p* = 0.05, respectively). There were no significant differences between Control + and cultures stimulated with rDiACT + VEGF-A and rDiFBAL + VEGF-A, respectively. However, there were significant differences between Control + and cultures stimulated with VEGF-A (*F* = 15.15; *df* = 4; *p* = 0.0059), rDiACT (*F* = 15.15; *df* = 4; *p* = 0.0123), and rDiFBAL (*F* = 10.14; *df* = 4; *p* = 0.0101). There were no significant differences between untreated cultures (Control) and cultures treated with VEGF-A, rDiACT, and rDiFBAL.

**Figure 4:**
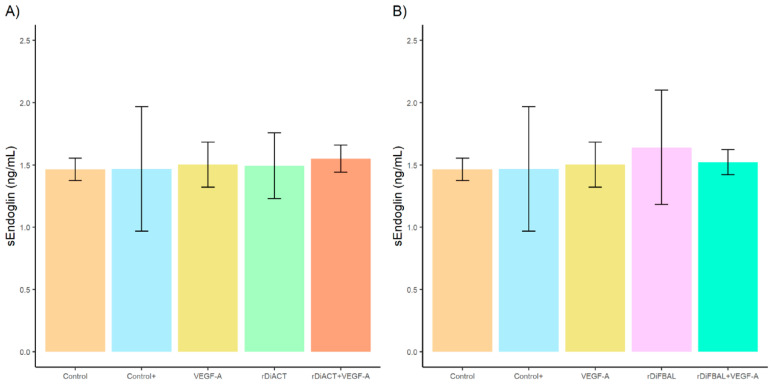
Effects of rDiACT (**A**) and rDiFBAL (**B**) on sEndoglin production in unstimulated cultures (Control) (■) and cultures stimulated with DiES + VEGF-A (Control +) (■), VEGF-A (■), rDiACT (■), rDiACT +VEGF-A (■), rDiFBAL (■), and rDiFBAL + VEGF-A (■). Results are expressed as the mean ± SD of 3 independent experiments.

**Figure 5:**
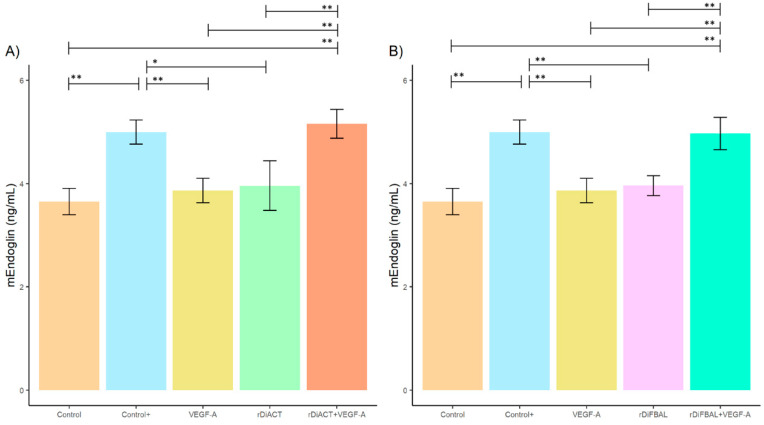
Effects of rDiACT (**A**) and rDiFBAL (**B**) on mEndoglin-2 production in unstimulated cultures (Control) (■) and cultures stimulated with DiES + VEGF-A (Control +) (■), VEGF-A (■), rDiACT (■), rDiACT +VEGF-A (■), rDiFBAL (■), and rDiFBAL + VEGF-A (■). Results are expressed as the mean ± SD of 3 independent experiments. The asterisk (*) and double asterisk (**) indicate significant differences with *p* < 0.05 and *p* < 0.01, respectively.

No significant differences were found between stimulated and unstimulated cultures with regard to sEndoglin production (Figure 4), but significant differences in mEndoglin production were observed between cell cultures stimulated with rDiACT + VEGF-A and rDiACT (*F* = 15.05; *df* = 4; *p* = 0.0057), VEGF-A (*F* = 15.05; *df* = 4; *p* = 0.0033), and untreated cultures (*F* = 15.05; *df* = 4; *p* = 0.001) (Figure 5). There were also significant differences between cultures stimulated with rDiFBAL + VEGF-A compared to cultures stimulated with rDiFBAL (*F* = 20.01; *df* = 4; *p* = 0.004), VEGF-A (*F* = 20.01; *df* = 4; *p* = 0.002), and untreated cultures (*F* = 20.01; *df* = 4; *p* = 0.0005) (Figure 4B). There were no significant differences between Control + and rDiACT + VEGF-A or rDiFBAL + VEGF-A, respectively, or between untreated cultures (Control) and cultures treated with VEGF-A, rDiACT and rDiFBAL. Moreover, there were significant differences between Control + and cultures stimulated with VEGF-A (*F* = 15.05; *df* = 4; *p* = 0.0083), rDiACT (*F* = 15.05; *df* = 4; *p* = 0.0148), and rDiFBAL (*F* = 20.01; *df* = 4; *p* = 0.0033).

### 3.2. Effect of Antigens on Cell Proliferation, Cell Migration, and Pseudocapillary Formation

**Figure 6:**
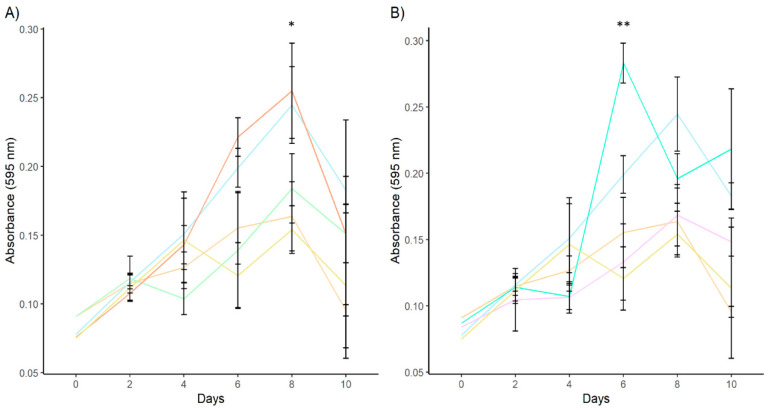
Effects of rDiACT (**A**) and rDiFBAL (**B**) on cell proliferation at 10 days in unstimulated cultures (Control) (■) and cultures stimulated with DiES + VEGF-A (Control +) (■), VEGF-A (■), rDiACT (■), rDiACT +VEGF-A (■), rDiFBAL (■), and rDiFBAL + VEGF-A (■). Results are expressed as the mean ± SD of 3 independent experiments. The asterisk (*) and double asterisk (**) indicate significant differences with *p* < 0.05 and *p* < 0.01, respectively.

In cell proliferation, all stimulated and unstimulated cell cultures show a typical cell growth curve with an increase in cell number between days 0 and 8 after stimulation and a decrease from day 10 after stimulation (Figure 6), except cell culture treated with rDiFBAL + VEGF that show continuous growth. When cells were treated with rDiACT + VEGF-A, there was a significant increase in the number of viable cells compared to cell cultures stimulated with rDiACT (*F* = 9.19; *df* = 4; *p* = 0.05), VEGF-A (*F* = 9.19; *df* = 4; *p* = 0.0064), and unstimulated cultures (*F* = 9.19; *df* = 4; *p* = 0.0124) at 8 days after stimulation. There were no significant differences between cultures stimulated with rDiACT + VEGF-A and DiES + VEGF-A (positive control) and between cultures stimulated with VEGF-A, rDiACT and untreated cells used as control. When cells were treated with rDiFBAL + VEGF-A, there was a significant increase in the number of viable cells compared to cultures treated with rDiFBAL (*F* = 25.63; *df* = 4; *p* < 0.0001), VEGF-A (*F* = 25.63; *df* = 4; *p* < 0.0001), and unstimulated cultures (*F* = 25.63; *df* = 4; *p* = 0.0002) at 6 days after stimulation. There were also no significant differences between cultures stimulated with rDiFBAL + VEGF-A and DiES + VEGF-A (positive control) and between cultures stimulated with VEGF-A, rDiFBAL and untreated cells used as control. The other days did not show relevant significant differences between proteins.

**Figure 7:**
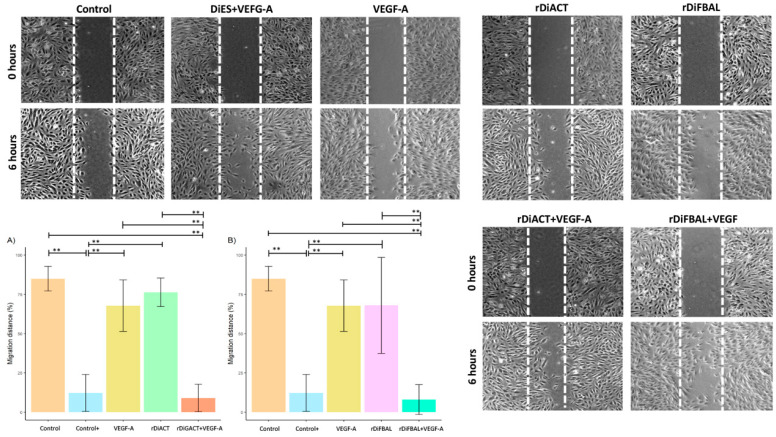
Representative images and effects of rDiACT (**A**) and rDiFBAL (**B**) on cell migration distance in unstimulated cultures (Control) (■) and cultures stimulated with DiES + VEGF-A (Control +) (■), VEGF-A (■), rDiACT (■), rDiACT +VEGF-A (■), rDiFBAL (■), and rDiFBAL + VEGF-A (■). Results are expressed as the mean ± SD of 3 independent experiments. Double asterisk (**) indicate significant differences with *p* < 0.01.

Regarding cell migration, there was a significant decrease in cell migration distance (Figure 7) in cultures stimulated with rDiACT + VEGF-A and rDiFBAL + VEGF-A compared to cultures stimulated with rDiACT (*F* = 53.48; *df* = 4; *p* < 0.0001) and rDiFBAL (*F* = 21.21; *df* = 4; *p* = 0.0001), respectively, to cultures stimulated with VEGF-A (*F* = 53.48; *df* = 4; *p* < 0.0001; and *F* = 21.21; *df* = 4; *p* = 0.0001, respectively) and to unstimulated cell cultures (*F* = 53.48; *df* = 4; *p* < 0.0001; and *F* = 21.21; *df* = 4; *p* < 0.0001, respectively). There were no significant differences between cultures stimulated with rDiACT + VEGF-A, rDiFBAL + VEGF-A, and positive control and between cultures stimulated with VEGF-A, rDiACT, rDiFBAL, and untreated cultures. Moreover, there were significant differences between Control + and cultures stimulated with VEGF-A (*F* = 53.48; *df* = 4; *p* < 0.0001), rDiACT (*F* = 53.48; *df* = 4; *p* < 0.0001), and rDiFBAL (*F* = 21.21; *df* = 4; *p* < 0.0001).

**Figure 8:**
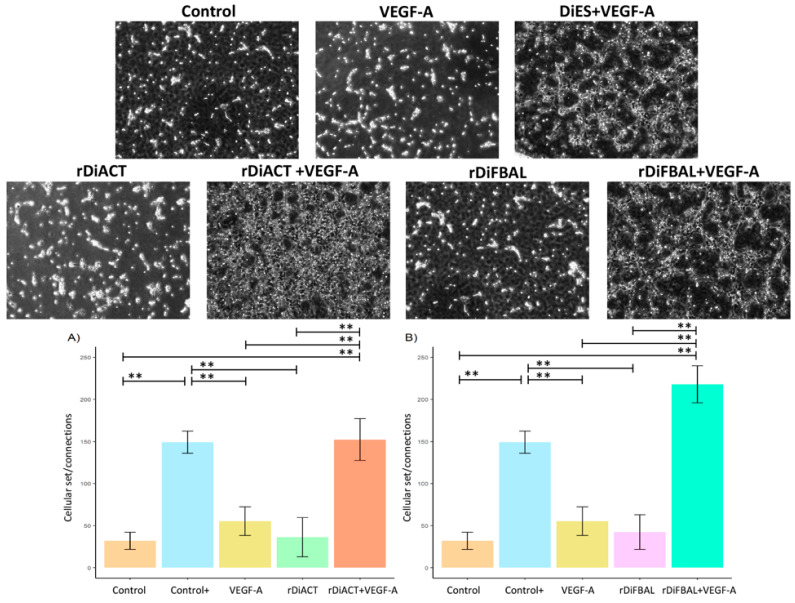
Representative images and effects of rDiACT (**A**) and rDiFBAL (**B**) on connections and cellular set in unstimulated cultures (Control) (■) and cultures stimulated with DiES + VEGF-A (Control +) (■), VEGF-A (■), rDiACT (■), rDiACT +VEGF-A (■), rDiFBAL (■), and rDiFBAL + VEGF-A (■). Results are expressed as the mean ± SD of 3 independent experiments. Double asterisk (**) indicate significant differences with *p* < 0.01.

The capacity for pseudocapillary formation was measured by analyzing the cell junctions and cell bodies originating from stimulated and unstimulated cell cultures (Figure 8). Pseudocapillary formation and cell junction/cell body ratio in rDiACT + VEGF-A-stimulated cultures were significantly increased compared to rDiACT-stimulated (*F* = 32.09; *df* = 4; *p* = 0.0001), VEGF-A-stimulated (*F* = 32.09; *df* = 4; *p* = 0.0005), and unstimulated (*F* = 32.09; *df* = 4; *p* < 0.0001) cell cultures. There were no significant differences between cultures stimulated with rDiACT + VEGF-A and DiES + VEGF-A. A similar observation was made in the case of cultures treated with rDiFBAL + VEGF-A, showing a significant increase compared to cultures stimulated with rDiFBAL (*F* = 67; *df* = 4; *p* < 0.0001), VEGF-A (*F* = 67; *df* = 4; *p* < 0.0001), and unstimulated cell cultures (*F* = 67; *df* = 4; *p* < 0.0001). Moreover, there were no significant differences between cultures stimulated with rDiACT + VEGF-A, rDiFBAL + VEGF-A, and DiES + VEGF-A and between cultures stimulated with VEGF-A, rDiACT, rDiFBAL, and untreated cultures. Moreover, there were significant differences between Control + and cultures stimulated with VEGF-A (*F* = 32.09; *df* = 4; *p* = 0.0007), rDiACT (*F* = 32.09; *df* = 4; *p* = 0.0001), and rDiFBAL (*F* = 67; *df* = 4; *p* = 0.0001).

## 4. Discussion

*Dirofilaria immitis* survives many years in the definitive host, and microfilariae can live for up to 2 years in the bloodstream [10]. Different pathways of action have been reported by which the parasite could evade the immune and inflammatory responses [27,30,31,32,33].

Through the excretory/secretory antigen of *D. immitis* adult worms (DiES), the parasite activates the production of PGE_2_ (vasodilator) through cyclooxygenase 2 (COX-2), participates in the decrease in monocyte transmigration through vascular endothelial cells [34], and activates the relaxation of the endothelium through the production of nitric oxide [9]. In addition, *D. immitis* interacts with the fibrinolytic system by binding to plasminogen and generating plasmin from excreted/secreted molecules and the antigen associated with the surface of *D. immitis*, reducing thrombi in order to not exacerbate the pathology of the host [32]. There are three proteins (GAPDH, galectin, and actin) of the excretory/secretory antigen and one (FBAL) associated with the surface of *D. immitis* that activate the fibrinolytic system, but, in the long term, they also activate the proliferation and migration of endothelial and muscle cells, which could explain the appearance of proliferative endoarteritis [29,35].

The angiogenic process is induced by processes such as endothelial inflammation, scarring, and hypoxia, among others, in order to increase blood flow to growing tissues, thereby expanding and remodeling the vascular system, which is linked to cellular processes such as proliferation, migration, survival, and endothelial morphogenesis [36]. In relation to heartworm disease, there are few studies that address this process. On the one hand, the antigenic extract of adult worms of *D. immitis* treated with doxycilin and rWSP are capable of promoting the antiangiogenic pathway in an in vitro model of endothelial cells, increasing the production of VEGF-A and VEGFR-1 and decreasing the capacity for pseudocapillary formation [12,24,25]. On the other hand, DiES and the antigen associated with the surface of the parasite promote the synthesis of proangiogenic factors, as well as cell proliferation and migration and the formation of pseudocapillaries [22].

The aim of this study was to analyze the angiogenic capacity of a protein present in the surface-associated antigen (actin) and another found within the excretory/secretory antigen of adult worms of *D. immitis* (FBAL) that activate the fibrinolytic system using an in vitro model of endothelial cells with which this and other processes have been studied, and this has helped to understand the parasite–host relationship [12,22,23,25,26,29,31,34,35].

First, the presence of VEGF-A and other proangiogenic (sVEGFR-2 and mEndoglin) and antiangiogenic (VEGFR-1/sFlt1 and sEndoglin) factors was analyzed in endothelial cells stimulated with the proteins rDiACT and rDiFBAL together with VEGF-A, a precursor of angiogenesis, creating a condition that simulates the consequence of the hypoxic situation. The results obtained show that rDiACT and rDiFBAL together with VEGF-A promote the production of VEGF-A and proangiogenic factors (sVEGFR-2 and mEndoglin) and not that of the antiangiogenic factors (VEGFR-1/sFlt1 and sEndoglin). In other studies, similar results were observed when analyzing their production in endothelial cells stimulated with molecules associated with the surface of *D. immitis* and DiES and VEGF-A [22], as opposed to cultures stimulated with the somatic extract of adult worms of *D. immitis* treated with doxycycline and rWSP under hypoxic conditions, in which mainly antiangiogenic factors were produced [12,24,25], which supports our results. In addition, there are other studies in relation to other filarial parasites such as *Wuchereria bancrofti* and *Brugia malayi*, other nematodes such as *Trichinella spiralis*, and other parasites (*Schistosoma mansoni* and *S. haematobium*, *Taenia solium*) where a similar mechanism has been observed in relation to their survival in their host [37,38].

Secondly, the cellular processes of cell proliferation and migration and the formation of pseudocapillaries, directly related to angiogenesis, were analyzed. Stimulation with rDiACT or rDiFBAL + VEGF-A produced an increase in cell proliferation and migration, as well as in the formation of pseudocapillaries, in our in vitro model of endothelial cells. These processes are linked to the growth, expansion, and remodeling of the vascular endothelium, which, as a consequence of the proangiogenic pathway, could be related to the survival of the parasite. Similar results have been observed in cell cultures stimulated with DiES and molecules associated with the surface of *D. immitis* [22] and not with the somatic extract of adult worms of *D. immitis* treated with doxycycline and rWSP under hypoxic conditions [12,25], where, in addition, the formation of pseudocapillaries was reduced [25].

## 5. Conclusions

The proteins rDiACT from the antigen of *D. immitis* surface and rDiFBAL from the excretory/secretory antigen molecules, together with VEGF-A, activate the proangiogenic pathway as well as the cellular processes of cell proliferation and migration, as well as the formation of pseudocapillaries, in an in vitro model of endothelial cells. These results show the angiogenic potential of both proteins as a survival mechanism of *D. immitis*, which may help us to better understand the parasite–host relationship in the vascular endothelium. The synergistic or antagonistic effect of both molecules can also be studied in this model or other related ones to delve deeper into the angiogenic processes aimed at parasite survival. Future in vivo studies in experimental animals and clinical cases with different symptoms or parasite loads, among others, could determine the importance of these in vitro results and the future of treatment strategies for heartworm disease.

## Data Availability

The raw data supporting the conclusions of this article will be made available by the authors without undue reservation.
